# Forces and Disease: Electrostatic force differences caused by mutations in kinesin motor domains can distinguish between disease-causing and non-disease-causing mutations

**DOI:** 10.1038/s41598-017-08419-7

**Published:** 2017-08-15

**Authors:** Lin Li, Zhe Jia, Yunhui Peng, Subash Godar, Ivan Getov, Shaolei Teng, Joshua Alper, Emil Alexov

**Affiliations:** 1 Department of Physics and Astronomy, Clemson University, Clemson, SC 29634 USA; 2 Department of Chemical Engineering, Clemson University, Clemson, SC 29634 USA; 3 Department of Biology, Howard University, Washington, DC 20059 USA

## Abstract

The ability to predict if a given mutation is disease-causing or not has enormous potential to impact human health. Typically, these predictions are made by assessing the effects of mutation on macromolecular stability and amino acid conservation. Here we report a novel feature: the electrostatic component of the force acting between a kinesin motor domain and tubulin. We demonstrate that changes in the electrostatic component of the binding force are able to discriminate between disease-causing and non-disease-causing mutations found in human kinesin motor domains using the receiver operating characteristic (ROC). Because diseases may originate from multiple effects not related to kinesin-microtubule binding, the prediction rate of 0.843 area under the ROC plot due to the change in magnitude of the electrostatic force alone is remarkable. These results reflect the dependence of kinesin’s function on motility along the microtubule, which suggests a precise balance of microtubule binding forces is required.

## Introduction

The ability to predict if genetic mutations cause disease or not has enormous potential to impact human health^1, 2^. Efforts to make these predictions to date have largely been done by assessing the effect of a genetic mutation on the coded protein’s stability and amino acid conservation^3, 4^. While these predictions have had some success based on genome-wide work^5^, when considering the disease-causing effects of mutations in particular protein families, other, more function specific, features may be more successful^6, 7^.

The kinesin superfamily of microtubule motor proteins is responsible for a diverse set of cell biological functions including intracellular transport, ciliary assembly, mitosis, meiosis, cytoskeletal morphology, and microtubule dynamics regulation^8, 9^. These functions depend on kinesin’s force generating and motile properties^10, 11^. Kinesins, for example, are particularly critical to the development of neurons due to their ability to transport intracellular cargos, including synaptic vesicles, mitochondria, and newly synthesized protein complexes from the endoplasmic reticulum near the nucleus in the cell body to the growing tips of axons and dendrites^12^. Kinesins enable elongated neurons, sometimes more than a meter long, to overcome physical limitations associated with long distance diffusion^13^.

To accomplish the diversity of functions that kinesins perform, there are 14 recognized and numbered families of kinesins^8, 14^, as well as numerous ungrouped, or orphan, kinesins^8^. Most members of the kinesin superfamily are microtubule plus end-directed motors^12^. Some notable exceptions include kinesin-13s, which are primarily involved in regulation of microtubule dynamics^15^ and move by diffusion^16^, and kinesin-14s, which are minus end-directed motors^17, 18^. Kinesin motor motility and pN-scale forces arise from structural changes in the neck linker subdomains^19, 20^ of kinesins upon hydrolysis of ATP^21^. However, these forces are not the only forces within kinesins that are critical to their function.

The forces of binding between a kinesin and the microtubule are additionally important to the motor’s processivity, which is a motility property determined by how far it moves along a microtubule before completely dissociating^13^. A single bound kinesin motor domain is in a state of force equilibrium in the absence of external loading, meaning that the sum of all forces between the microtubule and the kinesin must be zero. These forces are electrostatic and non-electrostatic forces, including hydrogen bonds and salt bridges, van der Waals forces, as well as others. However, because the charge of amino acids at the microtubule binding interface greatly affects the motility and microtubule-stimulated ATPase rate of kinesin^22^, the dominant force associated with binding is likely the electrostatic force. Electrostatic forces guide kinesin-1 to its binding site^23^ and allow it to follow a single protofilament^24, 25^. Electrostatic forces also likely underlie the diffusive motility of kinesin-8^26, 27^ and kinesin-13^16^.

Kinesins are critical to cell biology, so they are also important to many aspects of life, particularly to cell division and the nervous system. Genetic defects in kinesin motor domains that cause errors in cell division are likely embryonic lethal. Somatic defects are found in and are distributed throughout many kinesins, including the motor domains^28^. These defects are prevalent in endometrial cancer, lung squamous cell carcinoma, and melanoma^28^. However, somatic defects are generally unique to single samples making it difficult to discern their significance to the cancer^28^. Multiple congenital disorders are caused by non-synonymous single nucleotide polymorphisms (nsSNPs) in kinesin motor domains, including nsSNPs in the kinesin-1 family member KIF5A that cause parkinsonism^29^, peripheral neuropathy^29–32^, Charcot–Marie–Tooth disease type 2^33^, retinitis pigmentosa^29^, and spastic paraplegia^33–35^; nsSNPs in the kinesin-1 family member KIF5C and the kinesin-5 family member KIF11 that cause microcephaly^36, 37^; nsSNPs in the kinesin-10 family member KIF22 that cause lepto-spondyloepimetaphyseal dysplasia^38^; nsSNPs in the kinesin-3 family member KIF1A that cause spastic paraparesis and sensory and autonomic neuropathy type-2^39^; and nsSNPs in the kinesin-5 family member KIF11 that cause primary lymphedema and chorioretinal dysplasia^37^.

Because nsSNPs in kinesins tend to cause neurological genetic disorders and electrostatic forces between the kinesin motor domain and the microtubule are critical to multiple physiological properties of kinesins that could be particularly important in neurons, we hypothesized that the nsSNPs found in kinesin motor domains that greatly affect the electrostatic forces acting between kinesin and microtubules would strongly correlate to the nsSNPs causing human disease. To probe this hypothesis, we investigated the effect of known kinesin motor domain nsSNPs on the electrostatic force between kinesin and tubulin dimers using computational techniques. This study is based on 50 nsSNPs causing missense mutations in the motor domains of 10 different genes coding for proteins from 8 different kinesin families identified from dbNSFP^40^ and annotated as disease-causing using the Human Gene Mutation Database^41^ and ClinVar^42^ complemented with 11 nsSNPs that do not cause disease taken from 1000 genomes project^43^. The goal is to determine whether the changes in the electrostatic forces caused by mutations can be used to discriminate disease-causing mutations from those that do not cause human disease.

## Materials and Methods

### Selection of kinesin nsSNPs

The kinesin nsSNPs were downloaded from dbNSFP^40^ and missense mutations located in the coding regions of the kinesin motors domains with structures available in the PDB^44^ were selected. The Human Gene Mutation Database (HGMD)^41^ and ClinVar^42^ were used to identify disease-causing mutations. This resulted in the selection of 50 mutations in various kinesins.

A total of 11 nsSNPs with the allele frequency greater than 1% in the 1000 Genomes Project^45^ were identified and used as common, non-disease causing polymorphisms in the healthy individuals.

Note that significantly more disease-causing mutations were identified than non-disease-causing mutations, but the inclusion of mutations with allele frequency smaller than 1% may result in mutations with unknown physiological importance. The list of all the mutations for this study is provided in Supplementary Materials Table S1.

### Preparation of kinesin-tubulin structures

The 61 selected mutants come from 10 kinesin proteins representing 8 kinesin families (Table 1). High resolution structures, those with better than 5 Å resolution and having no mutation, were downloaded from the Protein Data Bank (PDB)^46^ for 5 of the 10 kinesin proteins. If multiple structures were available for the same kinesin in PDB, the structure with the highest resolution was selected for this work.

**Table 1:** Details of the 10 wild type kinesin structures used.

Kinesin family	Human protein name	PDB:	Template	Nucleotide state of motor	Sequence Similarity (%)	X-ray resolution (Å)	Ref.
Kinesin-1	KIF5A	Swiss model	3WRD.A – mouse kinesin 1 (KIF5C)	apo	91.2	2.9	71
Kinesin-1	KIF5C	Swiss model	5HNY.C – rat kinesin 1/Drosophila kinesin 14 chimera (KIF5C/NCD)	AMPPNP	97.8	6.3	72
Kinesin-3	KIF1A	Swiss model	2HXH.C – mouse kinesin 3 (KIF1A)	ADP	95.9	11	73
Kinesin-4	KIF21A	Swiss model	3ZFD.A – mouse kinesin 4 (KIF4)	AMPPNP	57.1	1.7	74
Kinesin-4	KIF27	Swiss model	3ZFD.A - mouse kinesin 4 (KIF4)	AMPPNP	52.5	1.7	74
Kinesin-5	KIF11	1II6.A		ADP		2.1	75
Kinesin-8	KIF18A	3LRE.A		ADP		2.2	68
Kinesin-9	KIF9	3NWN.A		ADP		2.0	44
Kinesin-10	KIF22	3BFN.A		ADP		2.3	44
Kinesin-13	KIF2C	2HEH.A		ADP		2.2	44

High resolution structures were not available in the PDB for the other 5 kinesin proteins. Note that for some proteins, including KIF1A and KIF5A, structures were available, however, either the resolution was too low or the structure had mutations introduced into it. In the cases without structure, SWISS-MODEL^47^ was used to build protein homology models from templates with high sequence similarities. The top model from SWISS-MODEL was selected to model the corresponding kinesin motor structures.

Some of the structures had missing heavy atoms. Profix^48^ was used to fix these structures.

NAMD^49^ was used to perform a 10,000-step energy minimization for each structure. In NAMD minimizations, the CHARMM^50^ force field and the Generalized Born (GB) implicit solvent model were used.

There were no structures of the human kinesin-human tubulin complex available in PDB. However, there were many other kinesin-tubulin complex structures available, and kinesins share the same microtubule binding site^22, 51^. Therefore, kinesin-tubulin complex structures were made using Chimera^52^ to align each kinesin (Table 1) to the human α1A/β3 tubulin dimer structure (PDB ID 5JCO)^53^, using a model of human kinesin-5 and a mammalian tubulin dimer docked into a 9.5- Å cryo-EM map (PDB ID 4AQW)^54^ as a template. The C-termini (E-hooks) were not modeled since their structures are not available in the corresponding PDB files.

Building complex structure via structural alignment of the backbone atoms resulted in atomic clashes at the binding interface. To remove these structural clashes introduced during the modeling process, the kinesin-tubulin complex structures underwent 2000 steps of energy minimization using the CHARMM36^55^ force field in CHARMM^56^ software in which only amino acid side chains were free to move because a 10 kcal·mol^−1^·Å^−1^ harmonic constraint was placed on all backbone atoms.

The nsSNP structures were generated based on the wild type structure for each kinesin using PDB2PQR^57^. The protonation states of titratable group were assumed to be standard, roughly corresponding to pH = 7.0. Since the kinesins considered in this work are cytoplasmic kinesins, the physiological pH is 7.0. Only the mutated residue was energy optimized; all other atoms were kept in the same position as in the wild type structure to isolate the direct effects of electrostatic forces.

### Force calculations

Electrostatic forces were calculated for each kinesin-tubulin complex using DelPhiForce^58^. The force reported is the net electrostatic force exerted on a kinesin by its tubulin dimer binding partner. The electrostatic force on each individual atom and residue, which is used to analyze the detailed force distribution on each kinesin, was also calculated with DelPhiForce.

The forces on each kinesin were calculated in two states: the bound state and the unbound state. The bound state was considered to be the equilibrium complex position, which was determined as described in “Preparation of kinesin-tubulin structures”. The unbound state was obtained by displacing the kinesin 5 Å from the tubulin in the direction along the line between the mass center of the kinesin and tubulin dimer. The dielectric constant for water and protein were set as 80 and 2, respectively; the resolution of the grid was set at 2 grids/Å; the perfil was set at 70; the ionic strength of the solvent was set at 0 (zero salt concentration was used to be consistent with our previous studies and to avoid the ambiguity associated with explicit ion binding). However, to check the sensitivity of results, parallel calculations were done at physiological salt concentration corresponding to ionic strength I = 0.15 M. The dipolar boundary condition was used in all cases. Information on these parameters is available in the DelPhi^59, 60^ manual (http://compbio.clemson.edu/downloadDir/delphi/delphi_manual.pdf).

The electrostatic force difference, ${\rm{\Delta }}\bar{F}$, was defined as the difference between the electrostatic forces exerted on wild type and the corresponding mutant kinesins.

1 $${\rm{\Delta }}\bar{F}={\bar{F}}_{{\rm{mut}}}-{\bar{F}}_{{\rm{wt}}}$$

where ${\rm{\Delta }}\bar{F}$ and are vector quantities with components Δ*F*_lat_ (lateral direction), Δ*F*_long_ (longitude direction), and Δ*F*_bind_ (binding direction).

The relative force difference Δ*F*_rel_ is defined as:

2 $${\rm{\Delta }}{F}_{{\rm{rel}}}=|{\bar{F}}_{{\rm{mut}}}-{\bar{F}}_{{\rm{wt}}}|/|{\bar{F}}_{{\rm{wt}}}|$$

The relative force difference in the binding direction Δ*F*_*bind,rel*_ is defined as

3 $${\rm{\Delta }}{F}_{{\rm{b}}{\rm{i}}{\rm{n}}{\rm{d}},{\rm{r}}{\rm{e}}{\rm{l}}}=|{\bar{F}}_{{\rm{b}}{\rm{i}}{\rm{n}}{\rm{d}},{\rm{m}}{\rm{u}}{\rm{t}}}-{\bar{F}}_{{\rm{b}}{\rm{i}}{\rm{n}}{\rm{d}},{\rm{w}}{\rm{t}}}|/|{\bar{F}}_{{\rm{b}}{\rm{i}}{\rm{n}}{\rm{d}},{\rm{w}}{\rm{t}}}|$$

where ${\bar{F}}_{{\rm{bind}},{\rm{mut}}}$ and ${\bar{F}}_{{\rm{bind}},{\rm{wt}}}$ are the components of the electrostatic force between the microtubule and the mutant and wild type kinesin in the binding direction, respectively.

## Results

### Electrostatic forces act between kinesin and tubulin

**Figure 1:**
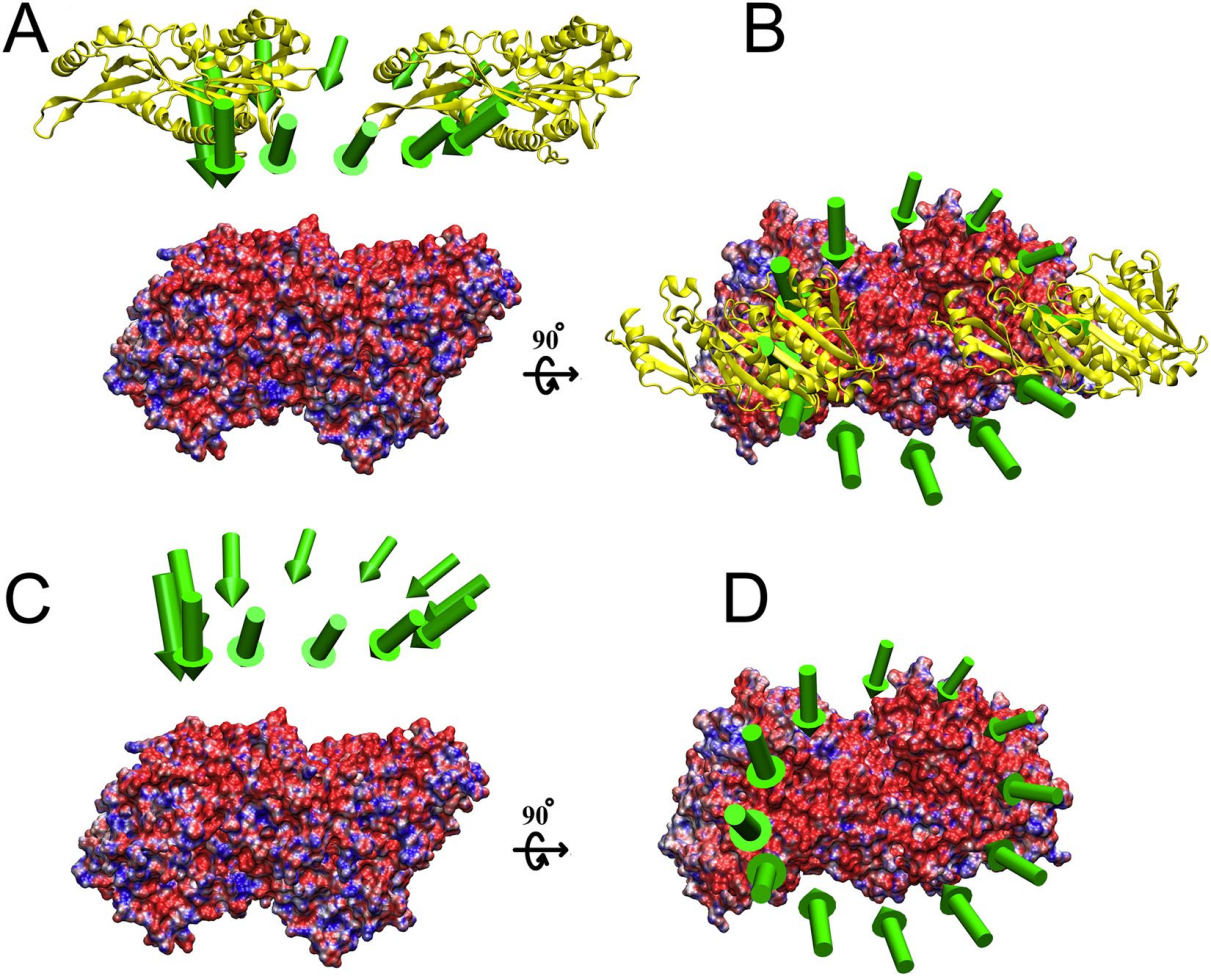
A funnel of electrostatic binding forces guides kinesin to the binding site on a tubulin dimer. The kinesin-13 structure (yellow) was shifted 20 Å away from its bound position and circled around the tubulin dimer (colored blue for positive surface charge and red for negative surface charge) along a circle with a radius of 40 Å. Every 30 degrees, the electrostatic force on the kinesin was calculated. These forces are represented by arrows (green) with their tail end located at the mass center of the kinesin in the 12 locations around the circle and their lengths proportional to the magnitude of the electrostatic force. (**A**) and (**C**) are the side views. (**B**) and (**D**) are the top views. In (**A**) and (**B**) the kinesin structure is shown at two positions for illustration of the range of displacement. In (**C**) and (**D**) the kinesin is hidden to provide clear view of the forces. In all frames, the total electrostatic forces were calculated using DelPhiForce and visualized with VMD^76^.

In our previous work, we demonstrated that the electrostatic forces on kinesin-5 form a binding funnel around the tubulin dimer^58^. A similar binding funnel was also found for dynein around the tubulin binding pocket^61^. In this work, we found that the binding funnel is common to kinesins, as shown for kinesin-13 as an example (Fig. 1), and that the electrostatic force guides the kinesin to the binding pocket of the tubulin. We obtained similar results for the other kinesins (Supplementary Material Table S1).

**Figure 2:**
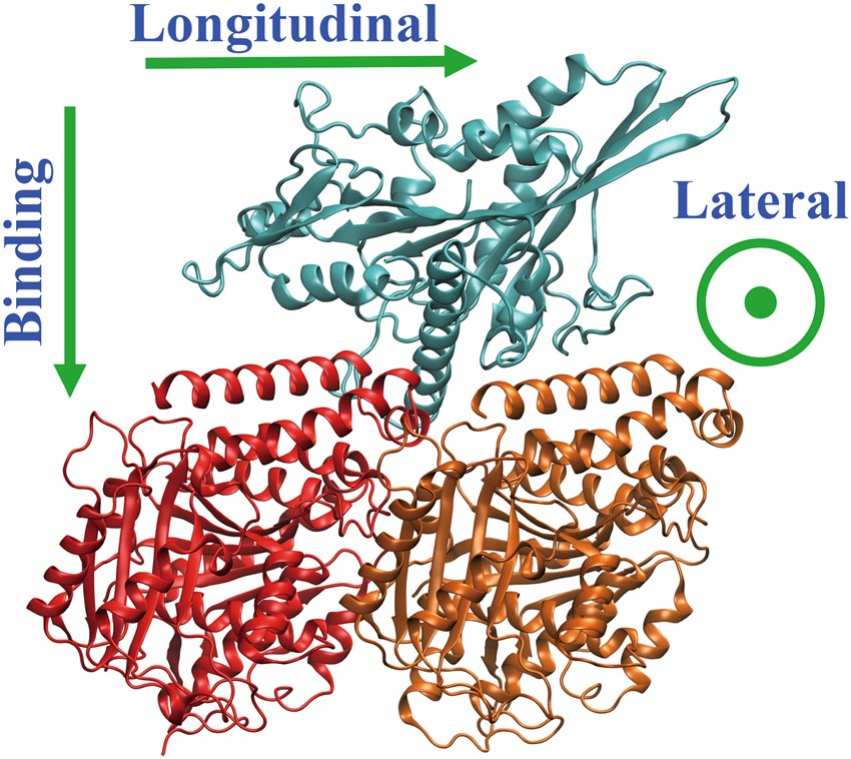
Definition of forces components. As an illustrative example, kinesin-3 family member KIF1A (light blue) is shown in the bound state on a tubulin dimer with α-tubulin (red) on the left side and β-tubulin (orange) on the right side. The “longitudinal” direction is along the microtubule, shown (green arrow) positive pointing toward the plus end. The “binding” direction is normal to the surface of the microtubule, shown (green arrow) positive toward the microtubule lumen. The “lateral” direction is around the microtubule, shown (green indicator) coming out of the page toward the reader.

Because the electrostatic force is a vector, $\bar{F}$, we examined its components in the longitudinal (*F*_long_), lateral (*F*_lat_), and binding (*F*_bind_) directions (Fig. 2) separately to further assess the role of electrostatic forces. We found that the magnitude of the mean electrostatic force, $|{\bar{F}}_{{\rm{avg}}}|$, for the 10 wild type kinesins used in this study in the bound state was 1,450 ± 170 pN (results for each kinesin are shown in Supplemental Material Table S1). Preforming the same calculations for unbound kinesins (at a displacement of 5 Å from the tubulin) resulted in an 87% decrease in $|{\bar{F}}_{{\rm{avg}}}|$ to 192 ± 56 pN. However, despite the large drop in magnitude, the direction of that mean force, and therefore the contribution of individual components, in the bound state was statistically indistinguishable from the unbound state (Table 2). The component of the mean electrostatic force in the binding direction, *F*_bind,avg_, contributed the most to the force magnitude (Table 2), and the components in the lateral, *F*_lat,avg_, and longitudinal, *F*_long,avg_, directions were not statistically different from zero (Table 2).

**Table 2:** Mean electrostatic force magnitude and direction.

Kinesin/Tubulin State	Electrostatic force magnitude (pN)	Components of the unit vector		
		Lateral	Binding	Longitudinal
Bound state	1450 ± 170	−0.16 ± 0.17	0.67 ± 0.08	0.10 ± 0.15
Unbound state	192 ± 56	−0.15 ± 0.17	0.77 ± 0.07	−0.01 ± 0.09

Note: Values are reported as mean ± standard error of the mean; n = 10 wild type kinesin proteins.

### Electrostatic forces and diseases

We calculated the electrostatic force differences, ${\rm{\Delta }}\bar{F}$ (Equation 1), of bound and unbound structures (Supplementary Material Table S1), where the force difference quantifies the difference in electrostatic force between the mutant and the corresponding wild type structure. Like the electrostatic force, force differences have three components in the longitudinal (Δ*F*_long_), lateral (Δ*F*_lat_), and binding (Δ*F*_bind_) directions (Fig. 2). Besides the force differences, we also calculated the relative force difference Δ*F*_rel_ (Equation 2). We found that mutations with larger values of relative force differences, Δ*F*_rel_, are more likely to cause disease (Supplementary Material Table S1).

**Figure 3:**
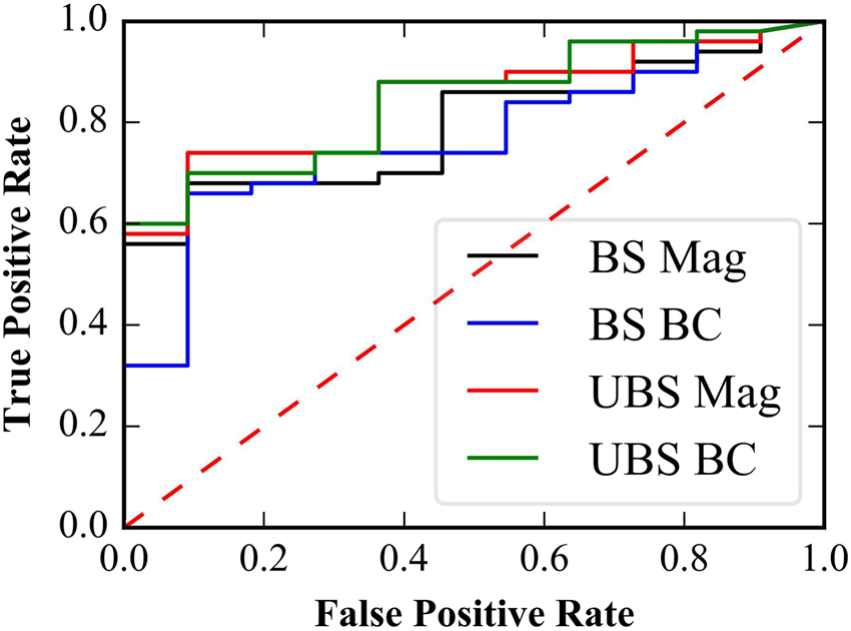
Magnitude of the electrostatic force difference, Δ*F*_rel_, can be used to predict whether a mutation is disease-causing. ROC plots are of Δ*F*_rel_ calculated in the bound state (BS Mag, black line), the component of force difference in the binding direction, Δ*F*_bind,rel_, in the bound state (BS BC, blue line), Δ*F*_rel_ in the unbound (UBS Mag, red line), and Δ*F*_bind,rel_ in the unbound state (UBS BC, green line). The areas below these four ROC curves are: 0.79, 0.77, 0.84, 0.84, respectively.

We quantified the result that large Δ*F*_rel_ tends to cause disease using Receiver Operating Characteristic (ROC) plots (Fig. 3). The area under an ROC plot indicates how well a descriptor, in this case Δ*F*_rel_, discriminates between two states, in this case whether a mutation is disease-causing or non-disease-causing. The area under an ROC plot of 1 indicates the descriptor can always discriminate between the states, and the area under a ROC plot of 0.5 (corresponding to the red dotted line in Fig. 3) indicates the descriptor is no better than random chance.

We found that Δ*F*_rel_ of unbound structures provided a better prediction of disease than Δ*F*_rel_ of bound structures because the areas under the unbound state ROC plots were 0.84 and 0.84 for Δ*F*_rel_ and Δ*F*_bind,rel_, respectively (Fig. 3) and the area under the bound state ROC plots were 0.79 and 0.77 for Δ*F*_rel_ and Δ*F*_bind,rel_, respectively (Fig. 3). We also noted that Δ*F*_rel_ performed slightly better than Δ*F*_bind,rel_ for structures in bound states (Fig. 3). We obtained similar results from ROC plots (Supplementary Material Figure S1) of electrostatic force calculations at an ionic strength of 0.15 M, indicating that ionic strength does not play a role in discriminating disease-causing from non-disease-causing mutations. Thus, in the rest of the manuscript, we focus on results obtained with I = 0 M. Since disease can be caused by either decreasing or increasing the wild type force, we did ROC using the absolute values of $|{\rm{\Delta }}\bar{F}|$ and |Δ*F*_bind_|, for both unbound and bound states, which resulted in similar as above performance; areas under ROC curve ranged from 0.72 to 0.75 (Supplementary Material Figure S2).

### Statistical analysis of electrostatic force components and disease-causing mutations

**Figure 4:**
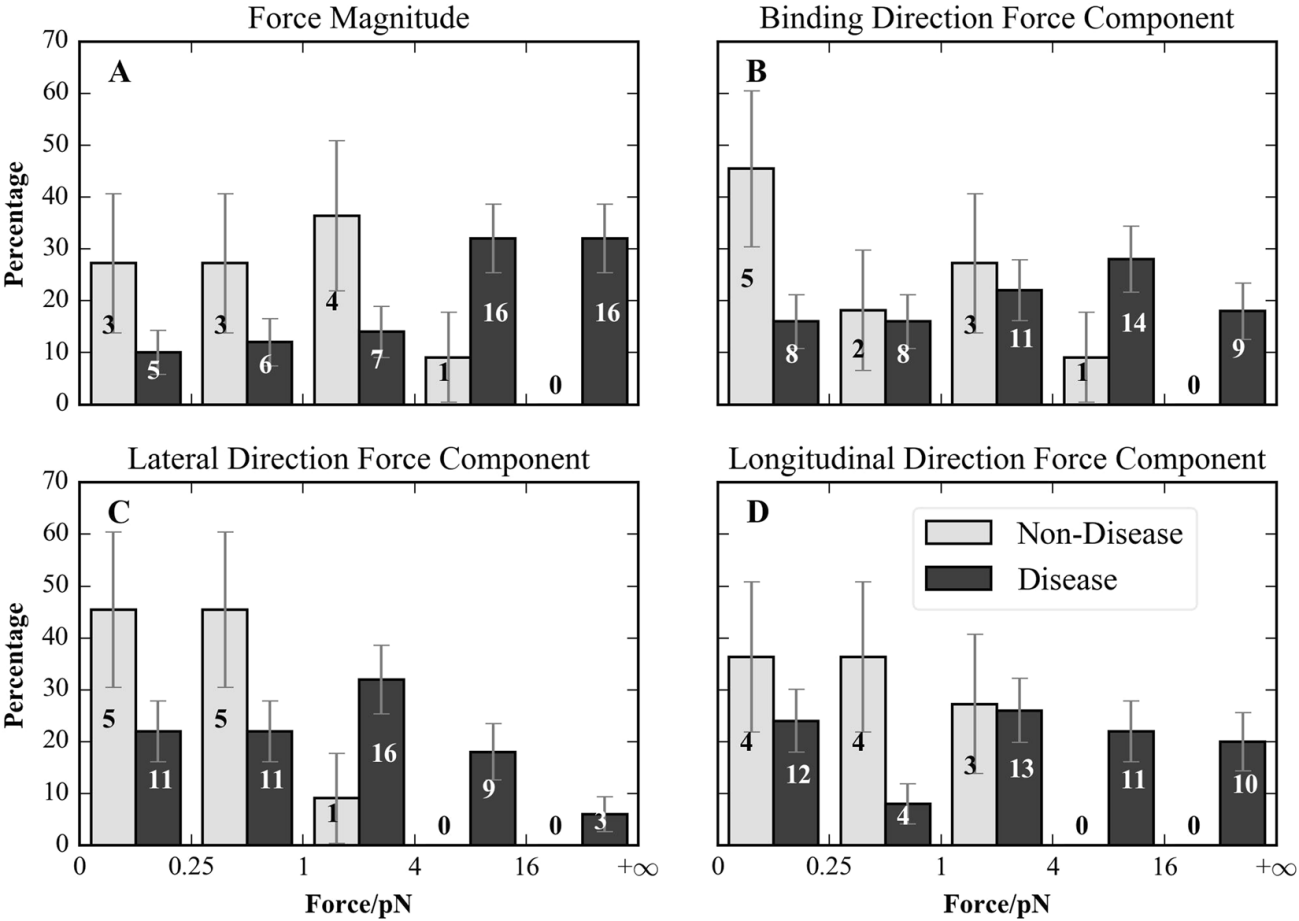
Whether a mutation causes a disease or not is correlated to the electrostatic force differences. Normalized histograms of disease-causing (black) and non-disease-causing (gray) mutations by electrostatic force difference when kinesin is in the unbound state for (**A**) $|{\rm{\Delta }}\bar{F}|$, (**B**) Δ*F*_bind_, (**C**) Δ*F*_lat_, and (**D**) Δ*F*_long_. Total mutation counts are labeled on each bar. Note that our dataset included a total of 50 disease-causing mutants and 11 non-disease-causing mutants. The error bars indicate the standard deviation.

We further investigated the unbound state’s $|{\rm{\Delta }}\bar{F}|\,\,$and its ${\rm{\Delta }}\bar{F}$’s components as predictors of whether a mutation is disease-causing or non-disease-causing using histograms (Fig. 4). We found that all mutations in our study with $|{\rm{\Delta }}\bar{F}| > 16$ pN led to disease and that only 9% of the non-disease causing mutations had $|{\rm{\Delta }}\bar{F}| > 4$ pN (Fig. 4A).

We also found that kinesins had a higher tolerance to Δ*F*_bind_ and Δ*F*_long_ than to Δ*F*_lat_. We found that only 9% of the non-disease-causing mutants had Δ*F*_lat_ > 1 pN while 36% had Δ*F*_bind_ > 1 pN and 27% had Δ*F*_long_ > 1 pN (Fig. 4B,C,D). We also noted that mutations causing Δ*F*_lat_ between 1 pN and 4 pN did a much better job distinguishing disease state because this range in Δ*F*_lat_ contains 35% of all disease-causing but only 9% of non-disease-causing mutants, which is statistically significantly different (*p*-value = 0.02), but this same range in Δ*F*_bind_ and Δ*F*_long_ had percentages of disease-causing and non-disease-causing that were statistically indistinguishable (Fig. 4B,C,D).

### Analysis of additional features that may be used to discriminate disease-causing and non-disease-causing mutations

We performed a statistical analysis of 23 features potentially affecting the pathogenicity of kinesin mutations using standard techniques (see Supplementary Material). By comparing the *p*-values of an F-regression analysis, we found that electrostatic force was the best predictor (Table 3). The other good predictors were the secondary structure of mutation position, change in binding free energy, and the location of mutation site (Table 3). The buried surface area, residue polarity, residue charge, etc., were not identified as significant features in predicting pathogenicity (Table 3).

**Table 3:** Statistical analysis of 23 possible features.

*Numerical Features*	*p*-value in f Regression	f Regression Score
Difference in total force at 5 Å distance	0.02	5.33
Absolute difference in binding force at 5 Å distance	0.03	5.19
Absolute difference in longitudinal force at 5 Å distance	0.06	3.68
Absolute difference in lateral force at 5 Å distance	0.07	3.40
Change in charge	0.12	2.51
Change in binding free energy	0.13	2.38
Change in buried surface area	0.23	1.49
Absolute difference in longitudinal force at bound state	0.24	1.42
Difference in total force at bound state	0.25	1.34
Absolute difference in longitudinal force at bound state	0.25	1.34
Absolute difference in binding force at bound state	0.28	1.18
Absolute difference in lateral force at bound state	0.30	1.09
Difference in longitudinal force at bound state	0.35	0.90
Difference in binding force at bound state	0.39	0.74
Change in folding free energy	0.40	0.71
Difference in lateral force at bound state	0.47	0.53
Difference in binding force at 5 Å Distance	0.57	0.33
Difference in lateral force at 5 Å Distance	0.60	0.28
Difference in longitudinal force at 5 Å Distance	0.69	0.16
***Categorical Features***	**Logistic Regression Coefficient**	
Change in polarity	0.46	
Residue on binding site	−0.01	
Residue exposure	−0.08	
Secondary structure of mutation residue	−0.23	

**Figure 5:**
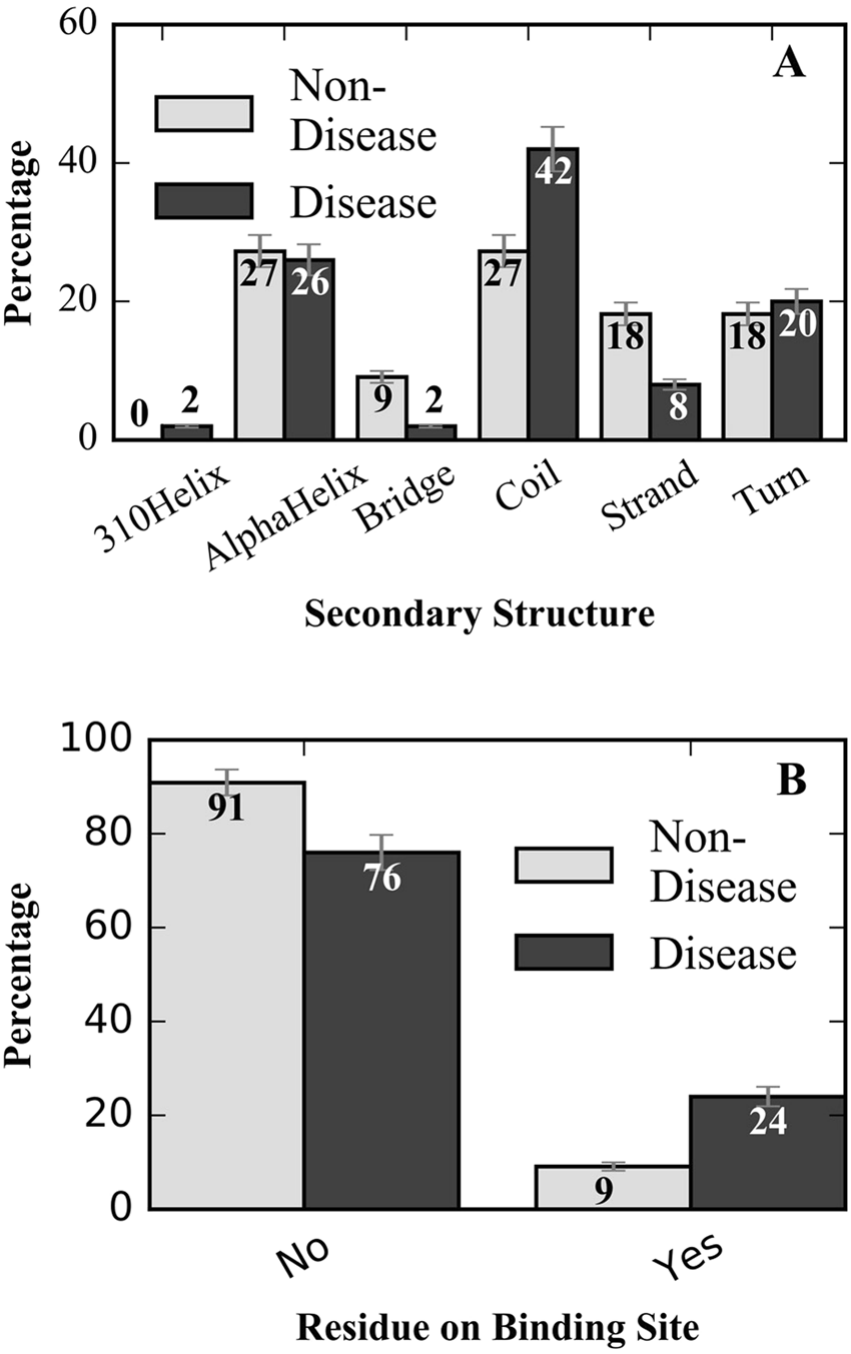
Location of the mutation is correlated to its likelihood of causing disease. (**A**) Histograms indicating which secondary structure the mutated residue is on for disease-causing and non-disease-causing mutants. (**B**) Histograms indicating whether the mutated residue is on the microtubule binding interface or not for disease-causing and non-disease-causing mutants.

We found that 88% of the disease-causing mutations occur in α-helices, coils, and turns (Fig. 5A). Only 31% of mutations located on strands caused disease, which is significantly fewer than the 61% and 53% disease-causing rates for mutations on coils and turns, respectively (Fig. 5A). Our data had few instances of mutations on 3–10 helices or salt bridges, therefore these mutations are not taken into further analysis.

Additionally, we noted that the disease-causing nature of a mutation was correlated to the function of the structure domain upon which it resides. 76% of mutations at the tubulin binding site were disease-causing (Fig. 5B), while mutations at other locations were disease-causing in only 46% of instances (Fig. 5B). Note that since this study focused on the kinesin-tubulin interaction, mutations on ATP binding site were not taken into further discussion.

## Discussion

We demonstrated that the changes of the electrostatic component of the force between kinesin and microtubule caused by amino acid mutations in the kinesin motor domain serve as a good discriminator between disease-causing and non-disease-causing mutations. 23 other features typically used by the computational community were also investigated, but we found them to be not as good predictors of disease state as the change of the electrostatic force. These results are remarkable because kinesin-related diseases may originate from nsSNPs causing effects within the motor domain not related to kinesin-microtubule binding. These effects may include disruption of nucleotide hydrolysis site because motility requires ATP hydrolysis^62^, proximity of the mutation to the location neck-linker-motor domain interaction site because motility requires neck linker docking^63, 64^, and motor domain structural stability because structure and function are closely correlated in structured proteins. Additionally, the kinesin family to which the mutated protein belongs could also be an important factor because certain families may have more critical cell or developmental biological function than others, and certain families may have fewer functional redundancies with other motors within the family than others^65^. Moreover, that our results show electrostatic force is a good discriminator between disease-causing and non-disease causing mutations suggests that there is steep electrostatic potential energy well about the kinesin docking location on the microtubule. Because the force is proportional to the spatial gradient of potential energy, small changes in the electrostatic energy potential result in large change in the force. Therefore, it is likely that electrostatic force is an even better discriminator than electrostatic energy potential.

**Figure 6:**
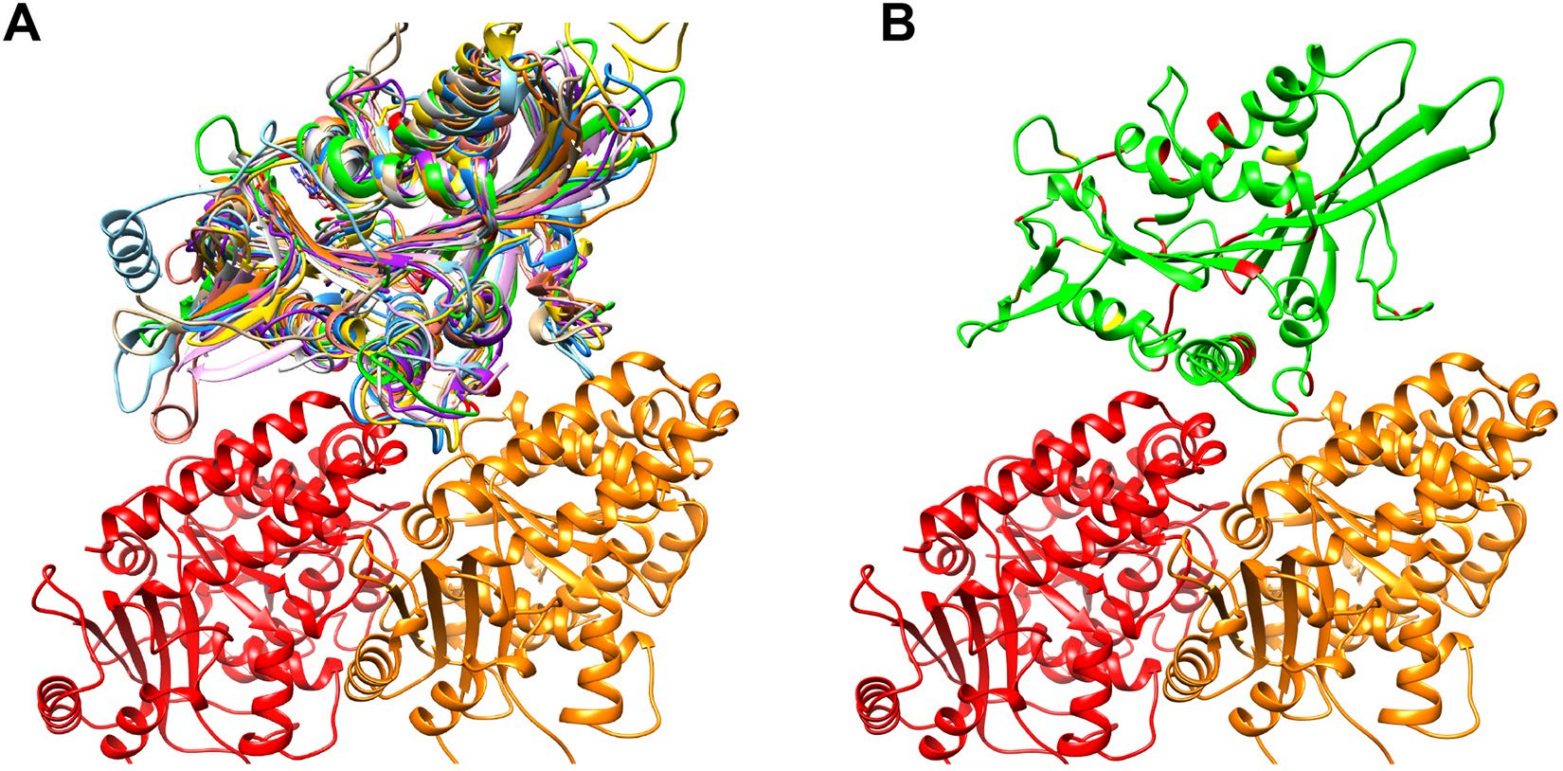
Mutations distribution map. (**A**) The structural alignment for all the kinesin-tubulin dimer structures studied, each color representing a different kinesin structure. (**B**) Mutations sites mapped on a representative kinesin structure (kinesin-3 family member, KIF1A). Red residues indicate disease-causing mutation sites and yellow residues indicate non-disease-causing mutation sites. α-tubulin (red) is on the left and β-tubulin (orange) is on the right in both panels.

We checked if our results were biased by the location of the mutations sites relative to the kinesin-microtubule binding interface by generating a representative kinesin motor domain-tubulin structure (Fig. 6). We note that the kinesin motor domains studied in this work have similar structures (Fig. 6A), and thus we use one (kinesin-3) to visualize the location of mutations sites (Fig. 6B). We found that there is no preference for disease-causing mutations to be at the binding interface while non-disease-causing mutations are away.

Recent studies indicate that positively charged residues on the kinesin motor domain strengthens its interaction with the microtubule, while negatively charged residues have an opposite effect^22, 23, 25, 66^. Consistent with these previous studies, we have found that most of the disease-causing mutations we studied involve charged residues. Our findings provide additional evidence for the importance of charged residues and electrostatics to kinesin motor domain microtubule binding. Furthermore, we found that Y274 and L248, which were previously identified as the top two most important uncharged residues for kinesin-microtubule binding^22, 66^, were also associated with disease-causing mutations in kinesin-3 family member KIF1A (L249Q) and in kinesin-1 family member KIF5A (Y276C). The correspondence between important previously identified charged and uncharged residues^22, 66^ and disease-association allows us to speculate that mutations at other positions identified as important in previous studies^22, 66^ including R346, K44, and K261, which do not appear in our database, are likely to be disease-causing.

Our key result is that if a mutation causes a $|{\rm{\Delta }}\bar{F}| > 4$ pN in the unbound state, then it is very likely to cause disease. Such a threshold roughly corresponds to 1 kcal/mol binding energy, an energy threshold that is widely used to discriminate disease-causing from non-disease-causing mutations^67^. Below we investigate a few particular mutants more closely, as illustrative examples, to understand our result a bit better.

**Figure 7:**
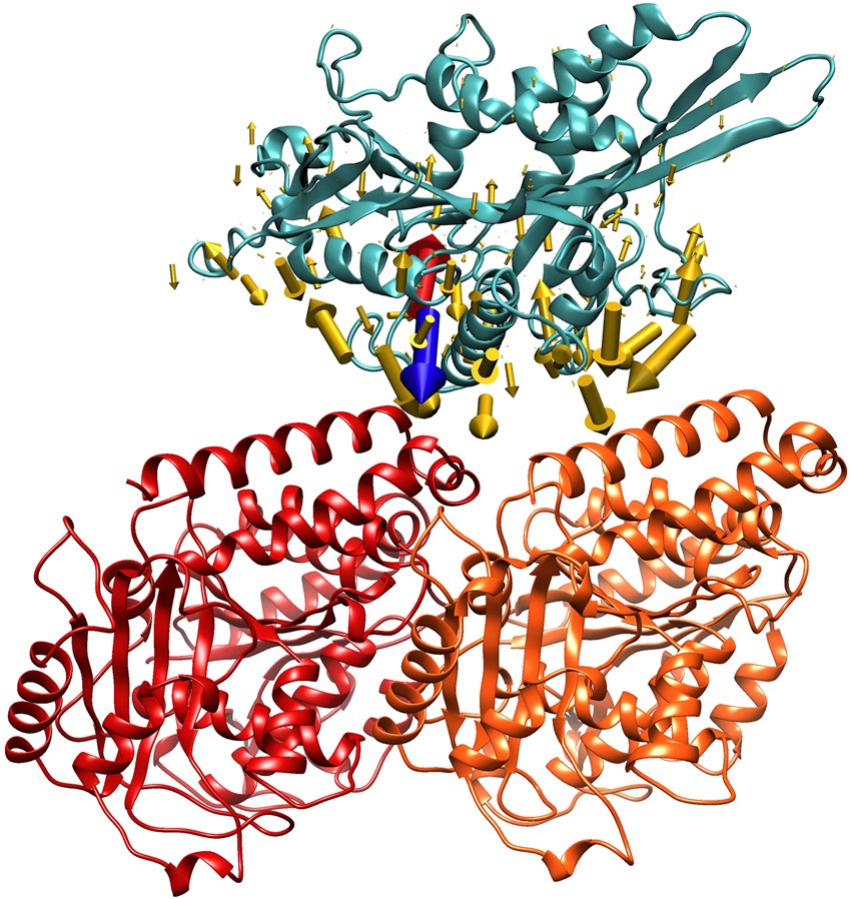
Forces on each residue of kinesin-3 show the large change in relative force due to the mutation. Kinesin-3 family member KIF1A (light blue) with the E253K mutation is shown bound to a tubulin dimer with α-tubulin (red) on the left side and β-tubulin (orange) on the right side. Most electrostatic forces (yellow arrows) on each residue of the kinesin-3 remain unchanged, but the force on residue 253 changes with the mutation, with both the force on wild type (red arrow) and the force on the mutant (blue arrow) shown.

First, we noted that kinesin-3 family member KIF1A E253K is charge reversal, from a negatively charged glutamic acid residue to a positively charged lysine residue, and it resulted in the largest $|{\rm{\Delta }}\bar{F}|$ (Supplementary Material Table S1) in both the bound and unbound states. We looked carefully at the magnitude and direction of the force on each amino acid in this kinesin-3 (Fig. 7). We found that the mutated amino acid lies close to the tubulin interface: the distance between the CA atom of E253 and the closest CA atom on tubulin is 9.6 Å. Because the mutation flips the charge of the residue and it is so close to the highly-charged tubulin interface, the large change in force we calculated was likely do the negative-to-positive charge reversal. The negatively charged E253 in wild type kinesin-3 opposes binding (Fig. 7 red arrow), and the positively charged K253 in the mutant kinesin-3 favors binding (Fig. 7 blue arrow), to the net negatively charged tubulin dimer. It is therefore not surprising that the enhanced binding due to this mutation causes spastic paraparesis and sensory and autonomic neuropathy type-2^39^ given that kinesin-3 drives long-distance transport in neuronal cells^9^.

Second, we noted that kinesin-8 family member KIF18A T273A mutant is the only non-disease causing mutant with a $|{\rm{\Delta }}\bar{F}| > 4$ pN; it had $|{\rm{\Delta }}\bar{F}|=4.92$ pN. We looked carefully at the location of this residue in the structure and found it to reside on an unstructured region (or at least one that is not in the PDB ID 3LRE structure)^68^ on the microtubule binding surface^22^, thus leading to a relatively large calculated $|{\rm{\Delta }}\bar{F}|$. However, the T273 is not highly conserved and the T273A does not change the motility of the kinesin in *in vitro* motility assays^22^. This could explain how this mutation is non-disease-causing despite relatively large $|{\rm{\Delta }}\bar{F}|$.

Third, we noted that the kinesin-1 family member KIF5A S203C mutant has a low $|{\rm{\Delta }}\bar{F}|=1.27$ pN in the unbound state (Supplementary Material Table S1), well below the discrimination threshold of 4 pN, but is disease-causing. We looked carefully at the location of this mutation, and found it is located in close proximity (5.5 Å) to the Mg^2+^ ion in the nucleotide binding site^69^. Specifically, S203 resides within a highly conserved sequence (NXXSSR, residues 199–204 of KIF5A) in switch I^30^, and it is thought to be important in recognizing the hydrolysis state of bound the nucleotide^70^. This could explain how this mutation causes a disease despite low $|{\rm{\Delta }}\bar{F}|$, highlighting our discrimination method’s limitation in finding all the true positive cases, particularly when mutations are unrelated to the kinesin-tubulin interaction.

While we did find that $|{\rm{\Delta }}\bar{F}| > 4$ pN in the unbound state is an excellent discriminator, we also found that the three components of the relative force difference, Δ*F*_long,rel_, Δ*F*_lat,rel_, and Δ*F*_bind,rel_, are also successful predictors, in their own right. These results suggest that the individual components of the binding force, particularly the lateral and longitudinal components, may be of critical importance for kinesin motility. It should be additionally noted that the magnitude of the electrostatic force is significantly (least 5-fold) larger in the binding direction than the other two directions (Table 1). Thus, it is likely to be less sensitive to the changes in magnitude than the other directions. If a mutation changes the force in the binding direction a given amount, kinesin may still bind to tubulin properly, however if the force in the lateral or longitudinal direction were changed by that same amount, it may be significantly more sensitive to the difference. It should be noted that the absolute value of the electrostatic force change was found to be the best discriminator. Thus, mutations strengthening the binding are equally likely to be disease-causing as mutations weakening it. This is consistent with previous studies on other systems, indicating that these systems are optimized and any deviation away from the wild type properties could be disease-causing^4, 67^.

Finally, it should be noted that this study considers the electrostatic component of the force acting between the kinesin and tubulin, not the total force. A kinesin motor domain that is not subjected to other external force, e.g. a cargo load, is at equilibrium on the microtubule. Therefore, at equilibrium, non-electrostatic forces must be acting at the tubulin-kinesin interface to balance out the large magnitude electrostatic forces we have calculated.

## Electronic supplementary material

Table 1S

Supplementary Information

## References

[1] Srinivasan S, Clements JA, Batra J (2016). Single nucleotide polymorphisms in clinics: Fantasy or reality for cancer?. Critical reviews in clinical laboratory sciences.

[2] Brookes AJ, Robinson PN (2015). Human genotype-phenotype databases: aims, challenges and opportunities. Nature Reviews Genetics.

[3] Leu C, Coppola A, Sisodiya SM (2016). Progress from genome-wide association studies and copy number variant studies in epilepsy. Current opinion in neurology.

[4] Kucukkal TG, Petukh M, Li L, Alexov E (2015). Structural and physico-chemical effects of disease and non-disease nsSNPs on proteins. Current opinion in structural biology.

[5] Alexov E, Sternberg M (2013). Understanding molecular effects of naturally occurring genetic differences. Journal of molecular biology.

[6] Kucukkal TG, Yang Y, Chapman SC, Cao W, Alexov E (2014). Computational and experimental approaches to reveal the effects of single nucleotide polymorphisms with respect to disease diagnostics. International journal of molecular sciences.

[7] Zhang, Z., Miteva, M. A., Wang, L. & Alexov, E. Analyzing effects of naturally occurring missense mutations. *Computational and mathematical methods in medicine* **2012** (2012). 10.1155/2012/805827 PMC3346971 22577471

[8] Endow SA, Kull FJ, Liu H (2010). Kinesins at a glance. J Cell Sci.

[9] Hirokawa N, Tanaka Y (2015). Kinesin superfamily proteins (KIFs): various functions and their relevance for important phenomena in life and diseases. Experimental cell research.

[10] Vale RD, Reese TS, Sheetz MP (1985). Identification of a novel force-generating protein, kinesin, involved in microtubule-based motility. Cell.

[11] Howard J, Hudspeth A, Vale R (1989). Movement of microtubules by single. Nature.

[12] Hirokawa N, Noda Y, Tanaka Y, Niwa S (2009). Kinesin superfamily motor proteins and intracellular transport. Nature reviews Molecular cell biology.

[13] Howard, J. *Mechanics of motor proteins and the cytoskeleton*. (Sinauer Associates, 2001).

[14] Lawrence CJ (2004). A standardized kinesin nomenclature. The Journal of cell biology.

[15] Desai A, Verma S, Mitchison TJ, Walczak CE (1999). Kin I kinesins are microtubule-destabilizing enzymes. Cell.

[16] Helenius J, Brouhard G, Kalaidzidis Y, Diez S, Howard J (2006). The depolymerizing kinesin MCAK uses lattice diffusion to rapidly target microtubule ends. Nature.

[17] Walker RA, Salmon ED, Endow SA (1990). The Drosophila claret segregation protein is a minus-end directed motor molecule. Nature.

[18] McDonald HB, Stewart RJ, Goldstein LS (1990). The kinesin-like ncd protein of Drosophila is a minus end-directed microtubule motor. Cell.

[19] Gittes F, Meyhöfer E, Baek S, Howard J (1996). Directional loading of the kinesin motor molecule as it buckles a microtubule. Biophysical Journal.

[20] Visscher K, Schnitzer MJ, Block SM (1999). Single kinesin molecules studied with a molecular force clamp. Nature.

[21] Hancock WO, Howard J (1999). Kinesin’s processivity results from mechanical and chemical coordination between the ATP hydrolysis cycles of the two motor domains. Proceedings of the National Academy of Sciences.

[22] Woehlke G (1997). Microtubule interaction site of the kinesin motor. Cell.

[23] Grant BJ (2011). Electrostatically biased binding of kinesin to microtubules. PLoS Biol.

[24] Ray S, Meyhöfer E, Milligan RA, Howard J (1993). Kinesin follows the microtubule’s protofilament axis. The Journal of cell biology.

[25] Li, L., Alper, J. & Alexov, E. Multiscale method for modeling binding phenomena involving large objects: application to kinesin motor domains motion along microtubules. *Scientific reports* **6** (2016). 10.1038/srep23249 PMC4796874 26988596

[26] Bormuth V, Varga V, Howard J, Schäffer E (2009). Protein friction limits diffusive and directed movements of kinesin motors on microtubules. Science.

[27] Jannasch A, Bormuth V, Storch M, Howard J, Schäffer E (2013). Kinesin-8 is a low-force motor protein with a weakly bound slip state. Biophysical Journal.

[28] Chandrasekaran G, Tátrai P, Gergely F (2015). Hitting the brakes: targeting microtubule motors in cancer. British journal of cancer.

[29] Goizet C (2009). Complicated forms of autosomal dominant hereditary spastic paraplegia are frequent in SPG10. Human mutation.

[30] Musumeci O (2011). A novel mutation in KIF5A gene causing hereditary spastic paraplegia with axonal neuropathy. Neurological Sciences.

[31] Schüle R (2008). SPG10 is a rare cause of spastic paraplegia in European families. Journal of Neurology, Neurosurgery & Psychiatry.

[32] Tessa A (2008). A novel KIF5A/SPG10 mutation in spastic paraplegia associated with axonal neuropathy. Journal of neurology.

[33] Crimella C (2012). Mutations in the motor and stalk domains of KIF5A in spastic paraplegia type 10 and in axonal Charcot–Marie–Tooth type 2. Clinical genetics.

[34] Kawaguchi K (2013). Role of kinesin-1 in the pathogenesis of SPG10, a rare form of hereditary spastic paraplegia. The Neuroscientist.

[35] Fichera M (2004). Evidence of kinesin heavy chain (KIF5A) involvement in pure hereditary spastic paraplegia. Neurology.

[36] Poirier K (2013). Mutations in TUBG1, DYNC1H1, KIF5C and KIF2A cause malformations of cortical development and microcephaly. Nature genetics.

[37] Ostergaard P (2012). Mutations in KIF11 cause autosomal-dominant microcephaly variably associated with congenital lymphedema and chorioretinopathy. The American Journal of Human Genetics.

[38] Min B-J (2011). Whole-exome sequencing identifies mutations of KIF22 in spondyloepimetaphyseal dysplasia with joint laxity, leptodactylic type. The American Journal of Human Genetics.

[39] Lee JR (2015). De novo mutations in the motor domain of KIF1A cause cognitive impairment, spastic paraparesis, axonal neuropathy, and cerebellar atrophy. Human mutation.

[40] Liu X, Wu C, Li C, Boerwinkle E (2016). dbNSFP v3. 0: A One‐Stop Database of Functional Predictions and Annotations for Human Nonsynonymous and Splice‐Site SNVs. Human mutation.

[41] Stenson PD (2009). The human gene mutation database: 2008 update. Genome medicine.

[42] Landrum MJ (2016). ClinVar: public archive of interpretations of clinically relevant variants. Nucleic acids research.

[43] Siva N (2008). 1000 Genomes project. Nature Biotechnology.

[44] Berman H, Henrick K, Nakamura H, Markley JL (2007). The worldwide Protein Data Bank (wwPDB): ensuring a single, uniform archive of PDB data. Nucleic acids research.

[45] Project G (2011). Variation in genome-wide mutation rates within and between human families. Nature genetics.

[46] Berman H, Henrick K, Nakamura H (2003). Announcing the worldwide protein data bank. Nature Structural & Molecular Biology.

[47] Guex N, Peitsch MC (1997). SWISS‐MODEL and the Swiss‐Pdb Viewer: an environment for comparative protein modeling. electrophoresis.

[48] Xiang Z (2006). Advances in homology protein structure modeling. Current Protein and Peptide Science.

[49] Nelson MT (1996). NAMD: a parallel, object-oriented molecular dynamics program. International Journal of High Performance Computing Applications.

[50] Vanommeslaeghe K (2010). CHARMM general force field: A force field for drug‐like molecules compatible with the CHARMM all‐atom additive biological force fields. Journal of Computational Chemistry.

[51] Mizuno N (2004). Dynein and kinesin share an overlapping microtubule‐binding site. The EMBO journal.

[52] Pettersen EF (2004). UCSF Chimera—a visualization system for exploratory research and analysis. Journal of Computational Chemistry.

[53] Vemu A (2016). Structure and dynamics of single-isoform recombinant Neuronal Human Tubulin. Journal of Biological Chemistry.

[54] Goulet A (2012). The structural basis of force generation by the mitotic motor kinesin-5. Journal of Biological Chemistry.

[55] Huang J, MacKerell AD (2013). CHARMM36 all‐atom additive protein force field: Validation based on comparison to NMR data. Journal of Computational Chemistry.

[56] Brooks BR (2009). CHARMM: The Biomolecular Simulation Program. Journal of Computational Chemistry.

[57] Dolinsky TJ, Nielsen JE, McCammon JA, Baker NA (2004). PDB2PQR: an automated pipeline for the setup of Poisson–Boltzmann electrostatics calculations. Nucleic acids research.

[58] Li, L., Chakravorty, A. & Alexov, E. DelPhiForce, a tool for electrostatic force calculations: Applications to macromolecular binding. *Journal of Computational Chemistry* (2017). 10.1002/jcc.24715 PMC5315605 28130775

[59] Li L (2012). DelPhi: a comprehensive suite for DelPhi software and associated resources. BMC biophysics.

[60] Li L, Li C, Zhang Z, Alexov E (2013). On the dielectric “constant” of proteins: smooth dielectric function for macromolecular modeling and its implementation in Delphi. Journal of chemical theory and computation.

[61] Li, L., Alper, J. & Alexov, E. Cytoplasmic dynein binding, run length, and velocity are guided by long-range electrostatic interactions. *Scientific reports* **6** (2016). 10.1038/srep31523 PMC4987762 27531742

[62] Coy DL, Wagenbach M, Howard J (1999). Kinesin takes one 8-nm step for each ATP that it hydrolyzes. Journal of Biological Chemistry.

[63] Cao, L. *et al.* The structure of apo-kinesin bound to tubulin links the nucleotide cycle to movement. *Nature communications* **5** (2014). 10.1038/ncomms6364 25395082

[64] Muretta JM (2015). The structural kinetics of switch-1 and the neck linker explain the functions of kinesin-1 and Eg5. Proceedings of the National Academy of Sciences.

[65] Goldstein LS (1991). The kinesin superfamily: tails of functional redundancy. Trends in cell biology.

[66] Li M, Zheng W (2011). Probing the Structural and Energetic Basis of Kinesin–Microtubule Binding Using Computational Alanine-Scanning Mutagenesis. Biochemistry.

[67] Petukh M, Kucukkal TG, Alexov E (2015). On Human Disease‐Causing Amino Acid Variants: Statistical Study of Sequence and Structural Patterns. Human mutation.

[68] Peters C (2010). Insight into the molecular mechanism of the multitasking kinesin‐8 motor. The EMBO journal.

[69] Atherton J (2014). Conserved mechanisms of microtubule-stimulated ADP release, ATP binding, and force generation in transport kinesins. Elife.

[70] Kikkawa M (2008). The role of microtubules in processive kinesin movement. Trends in cell biology.

[71] Morikawa M (2015). X‐ray and Cryo‐EM structures reveal mutual conformational changes of Kinesin and GTP‐state microtubules upon binding. The EMBO journal.

[72] Yamagishi M (2016). Structural Basis of Backwards Motion in Kinesin-1-Kinesin-14 Chimera: Implication for Kinesin-14 Motility. Structure.

[73] Kikkawa M, Hirokawa N (2006). High‐resolution cryo‐EM maps show the nucleotide binding pocket of KIF1A in open and closed conformations. The EMBO journal.

[74] Chang Q, Nitta R, Inoue S, Hirokawa N (2013). Structural basis for the ATP-induced isomerization of kinesin. Journal of molecular biology.

[75] Turner J (2001). Crystal structure of the mitotic spindle kinesin Eg5 reveals a novel conformation of the neck-linker. Journal of Biological Chemistry.

[76] Humphrey W, Dalke A, Schulten K (1996). VMD: visual molecular dynamics. Journal of molecular graphics.

