# Supplementary material for: Forces and Disease: Electrostatic force differences caused by mutations in kinesin motor domains can distinguish between disease-causing and non-disease-causing mutations
Source: Sci Rep. 2017 Aug 15;7:8237. doi: 10.1038/s41598-017-08419-7 (PMC5557957; doi:10.1038/s41598-017-08419-7)
Supplement: Supplementary file 2 — Supplementary Information [file 41598_2017_8419_MOESM2_ESM.pdf]

Forces and Disease: Electrostatic force differences caused by mutations in kinesin motor domains can distinguish between disease causing and non-disease-causing mutations

Lin Li[*,‡], Zhe Jia[*,‡], Yunhui Peng[‡], Subash Godar[‡], Ivan Getov[§], Shaolei Teng[||], Joshua Alper[†,‡], Emil Alexov[†,‡]

[‡] Department of Physics and Astronomy, Clemson University, Clemson, SC 29634

[§] Department of Chemical Engineering, Clemson University, Clemson, SC 29634

[||] Department of Biology, Howard University, Washington, DC 20059

Methods to analyze the 23 features

The contents in Table 3 are given as following. The numerical features are evaluated using f-regression versus the pathogenicity. Differences in forces are calculated by forces between microtubule and wild type motor protein subtract the forces of mutants. Absolute differences of forces are the absolute values of differences in forces. Change in charge (total charge), binding free energy, buried surface area during binding, and folding free energy are given by the properties of mutant subtract the values of wild type. The categorical features are scored using logistic regression based on their correlation to pathogenicity. Change in polarity means the change in polarity of mutated residue after mutation. The location of mutated residue is also taken into consideration, such as if the residue is on binding site, and its exposure to solvent (water). If any atom on the mutated residue is within 4Å to the microtubule in the bound state, it is considered as on the binding site. If the solvent accessible surface area (SASA) is larger than 10Å^2, it is marked as exposed. The secondary structure of the mutation residue is given by Stride webserver[1].

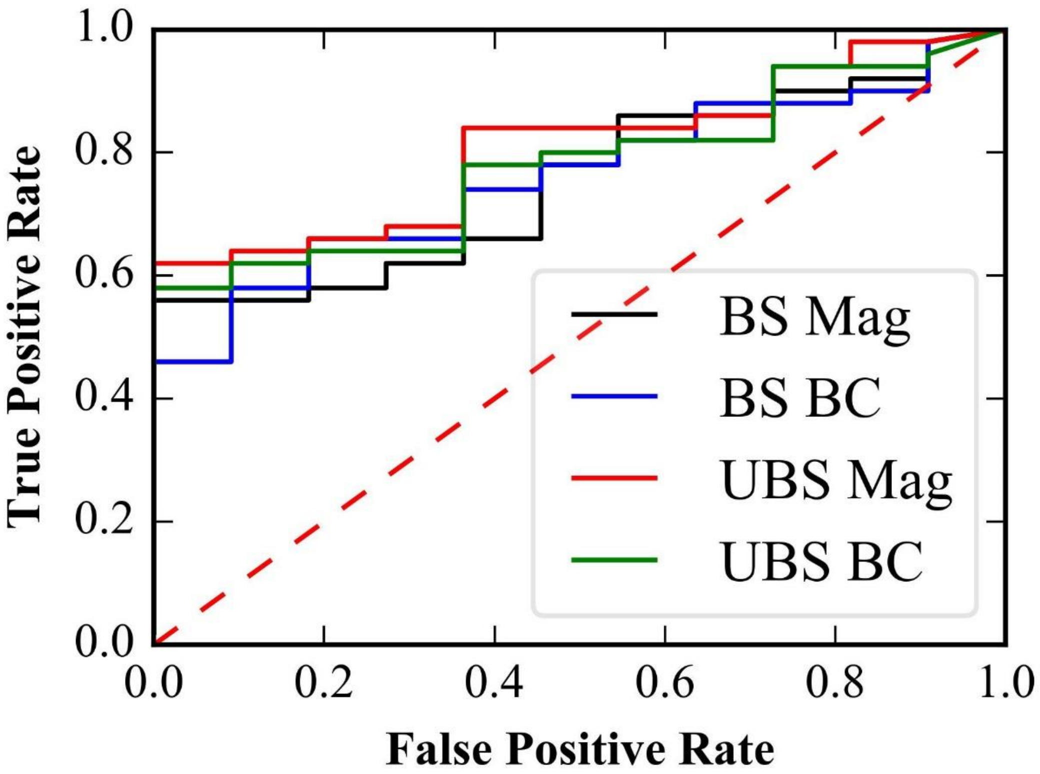

Figure S1. ROC plots are of ΔF_{rel} calculated in the bound state (BS Mag, black line), the component of force difference in the binding direction, $\Delta F_{\mathrm{bind,rel}}$, in the bound state (BS BC, blue line), ΔF_{rel} in the unbound (UBS Mag, red line), and $\Delta F_{\mathrm{bind,rel}}$ in the unbound state (UBS BC, green line). The corresponding forces were calculated at salt concentration corresponding to I=0.15M. The areas below these four ROC curves are: 0.79, 0.76, 0.80, 0.78, respectively.

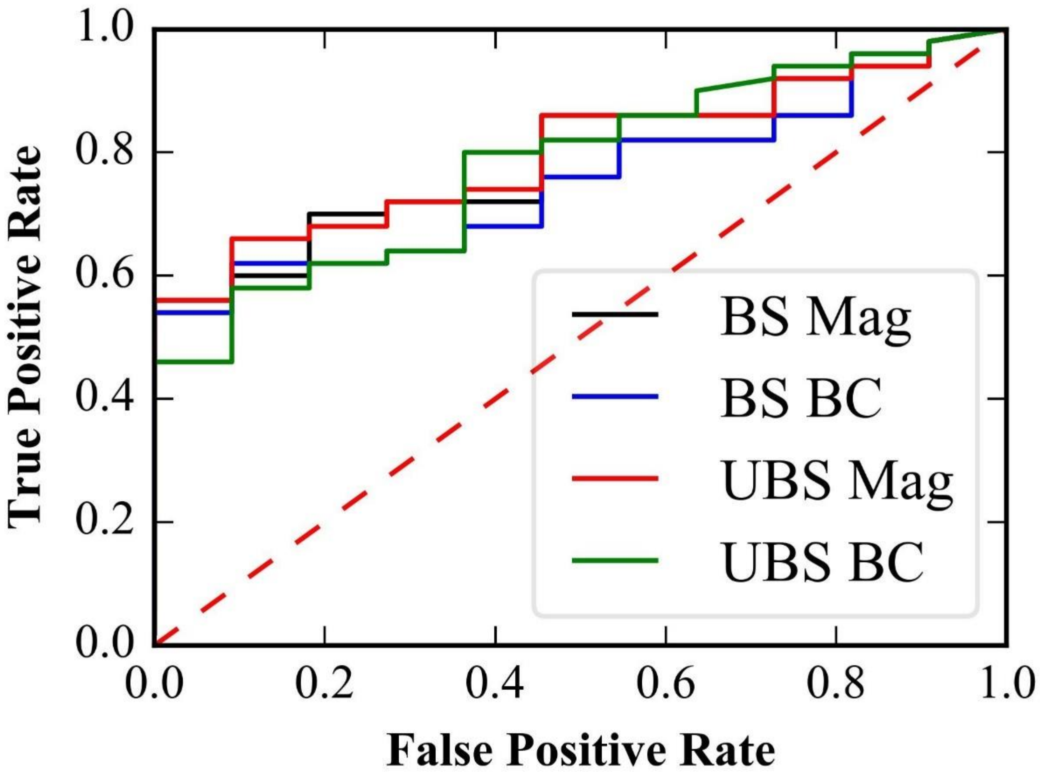

Figure S2. ROC plots are of $|\Delta\bar{F}|$ calculated in the bound state (BS Mag, black line), the component of force difference in the binding direction, $|\Delta F_{\text{bind}}|$, in the bound state (BS BC, blue line), $|\Delta\bar{F}|$ in the unbound (UBS Mag, red line), and $|\Delta F_{\text{bind}}|$ in the unbound state (UBS BC, green line). The corresponding forces were calculated without the present of salt. The areas below these four ROC curves are: 0.75, 0.75, 0.74, 0.72, respectively.

References:

1. Heinig, M.; Frishman, D., STRIDE: a web server for secondary structure assignment from known atomic coordinates of proteins. *Nucleic Acids Res* **2004,** *32* (Web Server issue), W500-2.
